# Comparison of the Quality of Sleep in Patients With Chronic Kidney Disease and End-Stage Renal Disease

**DOI:** 10.7759/cureus.23862

**Published:** 2022-04-05

**Authors:** Mehreen Mujahid, Kiran Nasir, Ruqaya Qureshi, Murtaza Dhrolia, Aasim Ahmad

**Affiliations:** 1 Nephrology, The Kidney Centre Post Graduate Training Institute, Karachi, PAK

**Keywords:** pitts-burgh sleep quality index, sleep disorders, hemodialysis, end stage renal disease, chronic kidney disease

## Abstract

Objective

In this study, we aimed to compare the quality of sleep between patients with (CKD) and those with end-stage renal disease (ESRD).

Methodology

We performed a cross-sectional study between August 2020 and January 2021. We included 240 patients, among which 178 (74.2%) were CKD patients and 62 (25.8%) were ESRD patients on maintenance hemodialysis (MHD). Demographic data were collected on a pre-designed proforma. The quality of sleep was evaluated using the Pittsburgh Sleep Quality Index (PSQI). PSQI assesses subjective sleep quality, sleep latency, sleep duration, habitual sleep efficiency, sleep disturbances, use of sleeping medication, and daytime dysfunction. A PSQI score >5 indicates poor sleep quality.

Results

Out of the 240 patients, 159 (66%) had poor sleep quality. We found a significant difference in mean PSQI scores between CKD and ESRD patients (9.6 ± 12.4 vs. 11.4 ± 3.9 respectively), indicating poorer sleep quality in ESRD patients as compared to those with CKD (p<0.001). In our study, among all comorbidities, poor sleep was significantly associated with ischemic heart disease (IHD) (p = 0.025), after adjusting for confounding factors.

Conclusions

Our study showed that two-thirds of the study population had poor sleep quality. ESRD patients suffered from more disturbed sleep as compared to CKD patients.

## Introduction

The growing burden of chronic kidney disease (CKD) all over the world including in Pakistan has grown to alarming proportions [1]. The global prevalence of CKD was estimated to be 9.1% (8.5-9.8) in 2017, with an increase of 29.3% (95% UI: 26.4-32.6) since 1990 [2]. Due to its chronic course, the condition has a negative impact on the quality of life (QOL) and quality of sleep.

Sleep disorders are highly prevalent in CKD patients, particularly among end-stage renal disease (ESRD) patients requiring maintenance hemodialysis (MHD) [3]. Good sleep is important for maintaining good health. Sleep helps in mental and physical relaxation, and it maintains the appropriate circadian rhythm and preserves energy for daily activities. Disturbed sleep causes fatigue and daytime sleepiness. An individual feels energetic, fit, and ready to meet new daily challenges after a good sleep [4].

Patients with CKD and ESRD suffer from various types of sleep disorders like restless leg syndrome (RLS), obstructive sleep apnea (OSA), insomnia, sleep-related breathing disorders (OSA, hypoventilation, and central sleep apnea), central disorders of hyper-somnolence, sleep-wake disorders, and parasomnias [5]. Poor QOL causes major problems in CKD and ESRD patients and greatly influences the course of the disease [6]. QOL has a direct impact on physical performance, emotional and physical wellbeing, and general health [7]. The pleasure of well-being and ability to function efficiently in daily life is compromised in patients with CKD and ESRD as compared to the general population [8].

The prevalence of poor sleep quality has been reported to be as high as 34-78% in patients with ESRD [9] and 14-57% in patients with CKD [10]. Despite sleep being an important part of the life of the patients, studies on the quality of sleep among CKD and ESRD patients in Pakistan are scarce. In light of this, we conducted this study to compare the quality of sleep in patients with CKD and ESRD.

## Materials and methods

This was a prospective cross-sectional study conducted at The Kidney Centre Post Graduate Training Institute (TKC-PGTI) in Karachi, Pakistan from July 23, 2020, to January 22, 2021, after obtaining ethical approval from The Kidney Centre Ethical Review Committee (TKC-ERC; Reference No. 80-NEPH-072019).

Inclusion criteria

Patients aged more than 18 years of both genders who signed a written informed consent form and who had been diagnosed with CKD for more than six months (CKD stage 2-5 pre-dialysis patients on regular follow-up at our nephrology clinic) and patients with ESRD on MHD at our hemodialysis unit were included.

Exclusion criteria

We excluded patients who had undergone major surgical interventions in the previous three months, those with cognitive impairment and/or dementia, physical disability, active psychosis, major hearing or a visual impairment, or malignancy. We also excluded kidney transplant patients.

Methodology

Demographic data [age, gender, marital status, education, profession, history of smoking and other addictions, duration of renal disease, other comorbidities like hypertension (HTN), diabetes mellitus (DM), ischemic heart disease (IHD)] and laboratory data [hemoglobin, urea, creatinine, calcium, phosphorus, albumin, uric acid, iron, ferritin, total iron-binding capacity (TIBC), hepatitis serology] were collected in a pre-designed proforma. BMI was calculated by the following formula: BMI = kg/m^2^ where kg is a person’s weight in kilograms and m^2^ is height in meters. The glomerular filtration rate (eGFR) of patients was calculated by using the CKD-EPI formula: GFR = 141 x min (serum creatinine/kappa, 1) alpha x max (serum creatinine/kappa, 1) -1.209 x 0.993 age x sex x race. For females, the following values were used: sex = 1.018; alpha = -0.329; kappa = 0.7. 

Quality of sleep

For the assessment of the quality of sleep, the Pittsburgh Sleep Quality Index (PSQI) was used. PSQI is an assessment tool for self-reporting of sleep problems. As the major spoken and written language in Pakistan is Urdu, we translated PSQI into Urdu (PSQI-U). With ERC approval, we performed a pilot study for the validation of PSQI-U on 10 non-medical, non-renal individuals. After the validation of PSQI-U, study subjects were requested to fill them in, which required 30-35 minutes. The patients who could not fill in the questionnaire themselves due to illiteracy were helped by the primary investigator (PI). PSQI contains 24 questions and seven components (first component: subjective sleep quality, second component: sleep latency, third component: sleep duration, fourth component: habitual sleep efficiency, fifth component: sleep disturbances, sixth component: use of sleeping medication, and seventh component: daytime dysfunction). In every domain of the scale, scoring is performed within a range of 0-3. The sum of the scores of these seven components constitutes the total index score. In this scale, the total value can be between 0 and 21 (interpretation: 0-5 = healthy sleep; 6-10 = poor sleep quality, and >10 = long-term sleep disturbance). A high score indicates that the sleep quality is impaired. Poor sleep quality is defined as a PSQI score >5.

Statistical analysis

Data were entered into and analyzed using SPSS Statistics version 20 (IBM, Armonk, NY). Mean ± standard deviation (SD) was computed for continuous variables (age, serum calcium, phosphorus, hemoglobin, iron, TIBC, creatinine, BMI, and PSQI score), while for categorical data, (gender, education, socioeconomic and marital status, status of hemodialysis, and poor quality of sleep) frequency with percentages was determined. Effect modifiers like age, gender, duration of disease, socioeconomic status, and education were controlled through stratification. Post-stratification chi-square was applied. A p-value less than or equal to 0.05 was considered statistically significant. Mean PSQI scores were compared between CKD and ESRD patients by using the independent t-test.

## Results

We included 240 patients of which 139 (57.9%) were males and 101 (42.1%) were female. The mean age of the participants was 47.6 ± 14.5 years. The majority of our patients were married (147, 61.3%), had primary education (117, 48.8%), and belonged to lower socioeconomic groups (135, 56.3%). HTN was the most prevalent comorbidity in our study population (217, 90.4%) (Table 1).

Out of the 240 patients, 159 (66%) had poor sleep quality. We found a significant difference between the mean PSQI scores of CKD patients and those of ESRD patients (9.6 ± 12.4 vs. 11.4 ± 3.9 respectively), indicating poorer sleep quality in ESRD patients as compared to patients with CKD (p<0.001) (Table 1).

**Table 1:** Comparison of study variables between CKD and ESRD patients CKD: chronic kidney disease; ESRD: end-stage renal disease; PSQI: Pittsburgh Sleep Quality Index; SD: standard deviation

Study variables		CKD, n = 178 (74.2%)	ESRD, n = 62 (25.8%)	Total study patients, n = 240	P-value
Gender, n (%)	Male	106 (59.6)	33 (53.2)	139 (57.9)	0.385
	Female	72 (40.4)	29 (46.8)	101 (42.1)	
Marital status, n (%)	Married	111 (62.4)	36 (58.1)	147 (61.3)	0.55
	Single	67 (37.6)	26 (41.9)	93 (38.7)	
Socioeconomic status, n (%)	Lower	98 (55.1)	37 (59.7)	135 (56.3)	0.722
	Middle	67 (37.6)	22 (35.5)	89 (37.1)	
	Upper	13 (7.3)	3 (25.8)	16 (6.7)	
Education, n (%)	Primary	93 (52.2)	24 (38.7)	117 (48.8)	0.002
	Secondary	52 (29.2)	34 (54.8)	86 (35.8)	
	Intermediate	27 (15.2)	4 (6.5)	31 (12.2)	
	Graduate & above	6 (3.4)	0 (0)	6 (2.5)	
Age in years, mean ± SD		47.5 ± 14.9	47.8 ± 13.2	47.6 ± 14.5	0.85
Smoker, n (%)		70 (70.7)	29 (29.3)	99 (41.3)	0.305
Hypertension, n (%)		168 (77.4)	49 (22.6)	217 (90.4)	<0.001
Diabetes mellitus, n (%)		98 (76.6)	30 (23.4)	128 (53.3)	0.365
Ischemic heart disease, n (%)		83 (74.1)	29 (25.9)	112 (46.7)	0.984
Body mass index, kg/m^2^,mean ± SD		21 ± 2.1	21 ± 2	8.9 ± 1.4	0.316
Hemoglobin, g/dL, mean ± SD		8.6 ± 1.4	9.2 ± 1.3	21.2 ± 2.1	0.085
Calcium, mg/dL, mean ± SD		8.3 ± 1.7	8.5 ± 1.8	8.3 ± 1.7	0.214
Phosphorus, mg/dL, mean ± SD		6.4 ± 2.4	6.1 ± 2.3	6.3 ± 2.5	0.374
Transferrin saturation, %, mean ± SD		45.1 ± 22.6	40.8 ± 22.6	44 ± 22.6	0.157
PSQI global score, mean ± SD		9.6 ± 12.4	11.4 ± 3.9	10.1 ± 3.7	<0.001

Table 2 shows the association between demographic characteristics of patients and PSQI global score. On stratification of data by age, gender, marital status, BMI, socioeconomic status, education status, smoking, DM, and HTN, we found that none of our variables except IHD had a significant impact on the association of stage of disease with quality of sleep. IHD as a comorbid condition had a significant association with PSQI global score (p = 0.025) (Table 3)

**Table 2:** Association of demographic characteristics of patients with PSQI global score PSQI: Pittsburgh Sleep Quality Index; SD: standard deviation

Demographic characteristics		PSQI global score		P-value
		1-5 = 24 (10%)	6-20 = 216 (90%)	
Gender, n (%)	Male	14 (58.3)	125 (57.9)	0.965
	Female	10 (41.3)	91 (42.1)	
Age, n (%)	18-35 years	3 (12.5)	58 (26.9)	0.217
	36-50 years	5 (20.8)	51 (23.6)	
	>50 years	16 (66.7)	107 (49.5)	
Marital status, n (%)	Married	17 (70.8)	130 (60.2)	0.31
	Single	7 (29.2)	86 (39.8)	
Education, n (%)	Primary	10 (41.7)	107 (49.5)	0.83
	Secondary	9 (37.5)	77 (35.6)	
	Intermediate	4 (16.7)	27 (12.5)	
	Graduate and above	1 (4.2)	5 (2.3)	
Socioeconomic status, n (%)	Low	16 (66.7)	119 (55.1)	0.151
	Middle	5 (20.8)	84 (38.9)	
	Upper	3 (12.5)	13 (6)	
Smoking status, n (%)	Smoker	6 (25)	93 (43.1)	0.088
	Non-smoker	18 (75)	123 (56.9)	
Body mass index, kg/m^2^, mean ± SD		20.8 ± 1.5	21.3 ± 2.2	0.404

**Table 3:** Association of clinical and laboratory parameters of patients with PSQI global score CKD: chronic kidney disease; ESRD: end-stage renal disease; PSQI: Pittsburgh Sleep Quality Index; SD: standard deviation

Clinical characteristics		PSQI global score		P-value
		1-5 = 24 (10%)	6-20 = 216 (90%)	
Hypertension, n (%)	Yes	24 (100)	193 (89.4)	0.093
	No	0 (0)	23 (10.6)	
Diabetes mellitus, n (%)	Yes	12 (50)	116 (53.7)	0.73
	No	12 (50)	110 (46.3)	
Ischemic heart disease, n (%)	Yes	6 (25)	106 (49.1)	0.025
	No	18 (75)	110 (50.9)	
Stage of renal disease, n (%)	CKD stage 2-5	19 (74.2)	159 (73.6)	0.555
	ESRD	5 (20.8)	57 (26.4)	
Hemoglobin, g/dL, mean ± SD		8.9 ± 1.7	8.9 ± 1.3	0.873
Calcium, mg/dL, mean ± SD		8.3 ± 1.5	8.3 ± 1.7	0.749
Phosphorus, mg/dL, mean ± SD		5.7 ± 2	6.4 ± 2.4	0.137
Transferrin saturation, %, mean ± SD		49.4 ± 23.5	43.4 ± 22.5	0.193

## Discussion

We found a significant difference in the quality of sleep between CKD patients and ESRD patients, and poor sleep quality was present in two-thirds of patients with CKD. Our study results are comparable with other studies [11,12].

Our study showed that out of 240 patients, 159 (66%) had poor sleep quality. In most studies, poor sleep quality was found in 57-78.5% of CKD and MHD patients [13,14]. In our study, the mean PSQI score was 9.6 ± 12.4 in CKD patients and 11.4 ± 3.9 in ESRD patients. Comparatively, the prevalence of poor sleep was 34-49% among ESRD patients on MHD [15] and 14-47% among patients with CKD [16,17]. The difference in the prevalence in different studies could be attributed to variations in patient selection, geographic location, and tools used for the assessment of the quality of sleep.

Very few studies have been conducted to compare the quality of sleep in CKD and ESRD patients, and most of them have shown varying results [18,19]. In our study, we found a significant difference in the quality of sleep between patients with CKD and those with ESRD. However, another study done in Pakistan did not find any significant difference in the quality of sleep between ESRD and CKD as per the PSQI global score. No significant correlation between eGFR and global PSQI score (r = -0.34, p = 0.80) in CKD patients was found, though poor sleep quality (PSQI ≥5) was present in 100 (65.8%) patients, which was comparable to our study [20].

Poor sleep is associated with cardiovascular diseases such as IHD and stroke [21,22]. We found a significant association between poor sleep and IHD (p = 0.025) among all comorbid conditions, after adjusting for confounding factors. Zhu et al. have observed an additive effect of poor sleep profile with respect to all-cause mortality and major adverse cardiovascular events (MACE) in the myocardial infarction with non-obstructive coronary arteries (MINOCA) population [23].

Our study has some limitations. Primarily, it was conducted at a single center. The number of patients in the two groups was not similar and this may have affected the results. Quality of sleep was assessed in a single time frame. Despite these limitations, our study, which was conducted at one of the largest dialysis units in the country, has proven that poor sleep quality is common in patients with CKD, especially those on MHD. ESRD patients on MHD experience poorer quality of sleep as compared to patients with CKD.

## Conclusions

The prevalence of CKD is on the rise globally, affecting people of all genders and ages. Poor sleep quality is common in patients with CKD and ESRD. Poor sleep affects QOL and increases the risk of adverse cardiovascular events. Clinicians treating these patients should routinely assess their quality of sleep and look for causative factors of poor sleep in these patients. More studies are needed to evaluate the impact of the quality of sleep on the risk of hospitalization, morbidity, and mortality in patients with CKD and ESRD.
